# Activation of PERK branch of ER stress mediates homocysteine-induced BK_Ca_ channel dysfunction in coronary artery via FoxO3a-dependent regulation of atrogin-1

**DOI:** 10.18632/oncotarget.17721

**Published:** 2017-05-09

**Authors:** Wen-Tao Sun, Xiang-Chong Wang, Shiu-Kwong Mak, Guo-Wei He, Xiao-Cheng Liu, Malcolm John Underwood, Qin Yang

**Affiliations:** ^1^ Division of Cardiology, Department of Medicine and Therapeutics, Institute of Vascular Medicine, Li Ka Shing Institute of Health Sciences, Institute of Innovative Medicine, The Chinese University of Hong Kong, Hong Kong, China; ^2^ TEDA International Cardiovascular Hospital, Chinese Academy of Medical Sciences, Tianjin, China; ^3^ Division of Cardiothoracic Surgery, Department of Surgery, The Chinese University of Hong Kong, Hong Kong, China

**Keywords:** cardiovascular risk factors, coronary circulation, endoplasmic reticulum stress, homocysteine, smooth muscle cell

## Abstract

The molecular mechanism of endoplasmic reticulum (ER) stress in vascular pathophysiology remains inadequately understood. We studied the role of ER stress in homocysteine-induced impairment of coronary dilator function, with uncovering the molecular basis of the effect of ER stress on smooth muscle large-conductance Ca^2+^-activated K^+^ (BK_Ca_) channels. The vasodilatory function of BK_Ca_ channels was studied in a myograph using endothelium-denuded porcine small coronary arteries. Primary cultured porcine coronary artery smooth muscle cells were used for mRNA and protein measurements and current recording of BK_Ca_ channels. Homocysteine inhibited vasorelaxant response to the BK_Ca_channel opener NS1619, lowered BK_Ca_ β1 subunit protein level and suppressed BK_Ca_ current. Inhibition of ER stress restored BK_Ca_ β1 protein level and NS1619-evoked vasorelaxation. Selective blockade of the PKR-like ER kinase (PERK) yielded similarly efficient restoration of BK_Ca_ β1, preserving BK_Ca_ current and BK_Ca_-mediated vasorelaxation. The restoration of BK_Ca_ β1 by PERK inhibition was associated with reduced atrogin-1 expression and decreased nuclear localization of forkhead box O transcription factor 3a (FoxO3a). Silencing of atrogin-1 prevented homocysteine-induced BK_Ca_ β1 loss and silencing of FoxO3a prevented atrogin-1 upregulation induced by homocysteine, accompanied by preservation of BK_Ca_ β1 protein level and BK_Ca_ current. ER stress mediates homocysteine-induced BK_Ca_ channel inhibition in coronary arteries. Activation of FoxO3a by PERK branch underlies the ER stress-mediated BK_Ca_ inhibition through a mechanism involving ubiquitin ligase-enhanced degradation of the channel β1 subunit.

## INTRODUCTION

Calcium-activated potassium (K_Ca_) channels contri-bute to the control of vascular tone. Being abundantly expressed in vascular smooth muscle cells, functional large-conductance K_Ca_ (BK_Ca_) channel is a tetrameric assembly of pore-forming α-subunits in complex with auxiliary β-subunits by which the channel activity is tightly regulated [1]. Activation of BK_Ca_channels opposes vasoconstriction via hyperpolarizing cell membrane thus limiting extracellular Ca^2+^ entry through voltage-dependent Ca^2+^ channels in smooth muscle cells [2]. Moreover, as a target site of endothelium-derived relaxing factors, i.e., nitric oxide (NO) [3, 4] and endothelium-derived hyperpolarizing factor [5, 6], BK_Ca_ channels are of importance in mediating endothelium-dependent relaxation in vasculatures. Disturbed function of BK_Ca_ channel has been found to be associated with various cardiovascular disorders such as hypertension [7] and diabetes [8].

Hyperhomocysteinemia is a prevalent and independent risk factor for atherosclerotic vascular disease and thromboembolism. In patients with coronary artery disease, plasma total homocysteine level is considered as a strong predictor of cardiovascular mortality [9]. Homocysteine causes vascular dysfunction through mechanisms of endothelial impairment including inhibition of endothelial NO synthase (eNOS) [10–12], induction of oxidative stress [13, 14], and activation of iNOS and arginase [15], etc. Previous studies reported the inhibitory effect of homocysteine on smooth muscle BK_Ca_ channels. The BK_Ca_ current was observed to be significantly suppressed in animal and human vascular smooth muscle cells subjected to homocysteine exposure [16, 17]. Nevertheless, the mechanisms by which homocysteine inhibits BK_Ca_ channels remain poorly studied.

Recent studies concerning the role of endoplasmic reticulum (ER) stress in vascular dysfunction highlighted endothelial mechanism. ER stress causes inhibition of eNOS-NO pathway and increases production of endothelium-derived contracting factors [18–20]. In homocysteine-induced endothelial dysfunction, ER stress was found to be involved in NADPH activation and reactive oxygen species (ROS) production as well as NF-κB activation [21]. We recently demonstrated that ER stress induced by homocysteine compromises endothelial function through suppression of intermediate- and small-conductance K_Ca_ (IK_Ca_ and SK_Ca_) channels [22]. Whether ER stress inhibits smooth muscle BK_Ca_ channels and BK_Ca_-mediated vasodilatory function so far remains uninvestigated.

Forkhead box o transcription factors (FoxOs) serve essential roles in maintaining vascular stability with significance in differentiation, proliferation, and survival of endothelial and smooth muscle cells [23, 24]. The finding that FoxOs may negatively regulate eNOS expression [25] supports a link between endothelial dysfunction and FoxOs dysregulation. Recent studies demonstrated that increase of FoxO3a transcriptional activity contributes to BK_Ca_ channel dysfunction in vessels of diabetic animals, which was believed to be mediated by muscle-specific E3 ubiquitin ligase, i.e., atrogin-1 [8, 26]. It was reported that FoxO activity is modulated by ROS [27]. Nevertheless, whether FoxOs respond to ER stress and how the response alters vascular dilator function are barely studied.

The present study aimed to investigate whether ER stress is involved in homocysteine-induced BK_Ca_ channel inhibition in coronary arteries. Further exploration of the molecular determinants of homocysteine-induced BK_Ca_ channel inhibition aimed to advance our understanding of the cellular mechanisms of ER stress signaling in vascular disorders associated with hyperhomocysteinemia.

## RESULTS

### ER stress mediates homocysteine-induced loss of dilatory function of BK_Ca_ channels in PCAs

**Figure 1:**
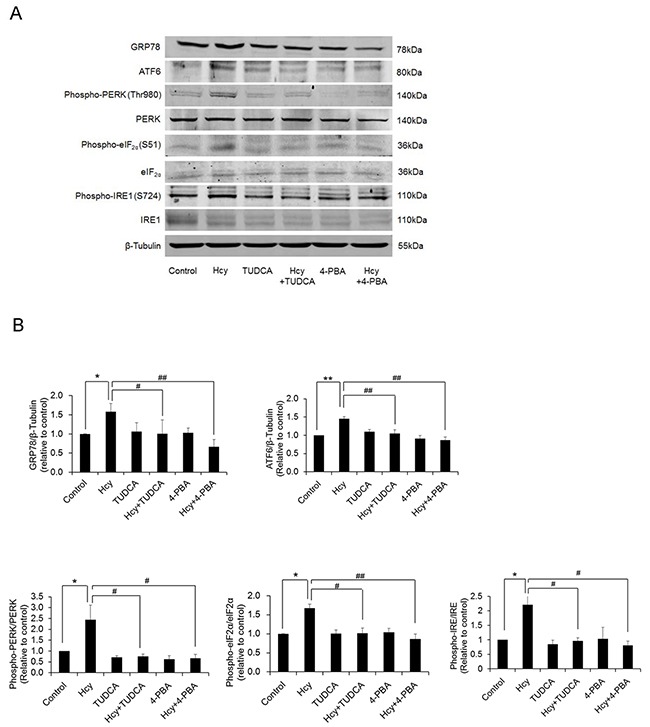
Homocysteine exposure induces ER stress in PCASMCs **(A)** representative blots of ER stress molecules; **(B)** expression levels of ER stress molecules from 4 independent experiments, each obtained from cell isolates of different heart. *p<0.05, **p<0.01; ^#^p<0.05, ^##^p<0.01. Hcy: homocysteine, TUDCA and 4-PBA: ER stress inhibitors.

**Figure 2:**
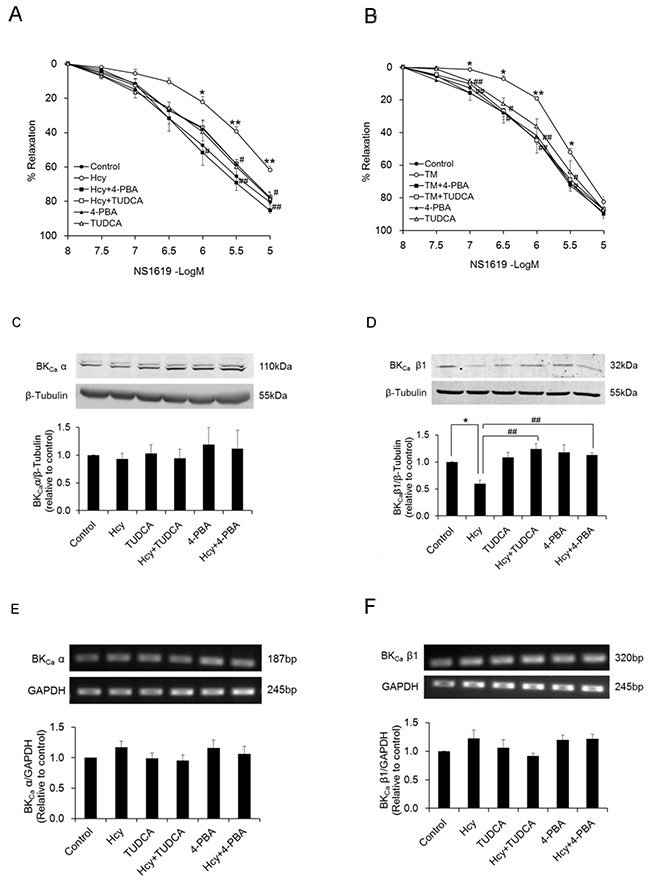
Vasorelaxant responses of PCAs to the BK_Ca_ channel activator NS1619 are attenuated after exposure to homocysteine (A, n=8) and tunicamycin (B, n=8) Treatment with ER stress inhibitors, either 4-PBA or TUDCA preserves the relaxant response in both homocysteine- (**A**) and tunicamycin- (**B**) exposed arteries. Homocysteine exposure lowers the protein level of BK_Ca_ β1 subunit in PCASMCs (n=5) whereas shows no significant effect on the protein content of α subunit (n=8) (**C** & **D**). Inhibition of ER stress during homocysteine exposure restores BK_Ca_ β1 protein level (**D**). Homocysteine does not alter mRNA expression levels of both α and β1 subunits of BK_Ca_ (**E** & **F**, n=7). N indicates the number of independent experiments, each obtained from vessels (a & b) or cell isolates (c-f) of different heart. *p<0.05, **p<0.01 vs. control; ^#^p<0.05, ^##^p<0.01 vs. Hcy or TM. Hcy: homocysteine, TM: tunicamycin.

Homocysteine induced ER stress in PCASMCs, evidenced by increased expression of ER stress molecules GRP78, ATF6 and enhanced phosphorylation of PERK, eIF2α, and IRE1. Both 4-PBA and TUDCA effectively inhibited ER stress in homocysteine-exposed PCASMCs (Figure 1). The BK_Ca_ channel activator NS1619 induced dose-dependent relaxation in endothelium-denuded PCAs. Exposure to homocysteine for 24 h did not alter the resting force of coronary arteries and there were no significant differences among groups with regard to U46619-induced pre-contraction (data not shown), whereas the NS1619-induced vasorelaxation was attenuated (R_max_: 61.9±2.3% vs. 80.6±3.6% in control, p<0.01), which was restored by ER stress inhibitors, 4-PBA (85.4±1.7%) and TUDCA (77.8±3.3%) (p<0.05 vs. homocysteine) (Figure 2A). In addition, exposure of PCAs to a chemical ER stress inducer tunicamycin also significantly attenuated the vasorelaxant response to NS1619 and such attenuation was prevented by either 4-PBA or TUDCA (Figure 2B). In arteries without homocysteine or tunicamycin exposure, the ER stress inhibitor itself did not alter BK_Ca_ channel-mediated vasorelaxation (Figure 2A & 2B).

### Homocysteine reduces protein level of BK_Ca_ β1 subunit in PCASMCs via an ER stress-dependent mechanism

Homocysteine exposure lowered the protein level of β1 subunit of BK_Ca_ in PCASMCs whereas showed no significant effect on the protein content of α subunit (Figure 2C & 2D). Inhibition of ER stress with either TUDCA or 4-PBA during homocysteine exposure preserved the protein level of BK_Ca_ β1 (Figure 2D). Exposure to homocysteine did not alter the mRNA expression of either α or β1 subunit of BK_Ca_ in PCASMCs (Figure 2E & 2F).

### PERK activation in response to ER stress mediates homocysteine-induced BK_Ca_ channel dysfunction

**Figure 3:**
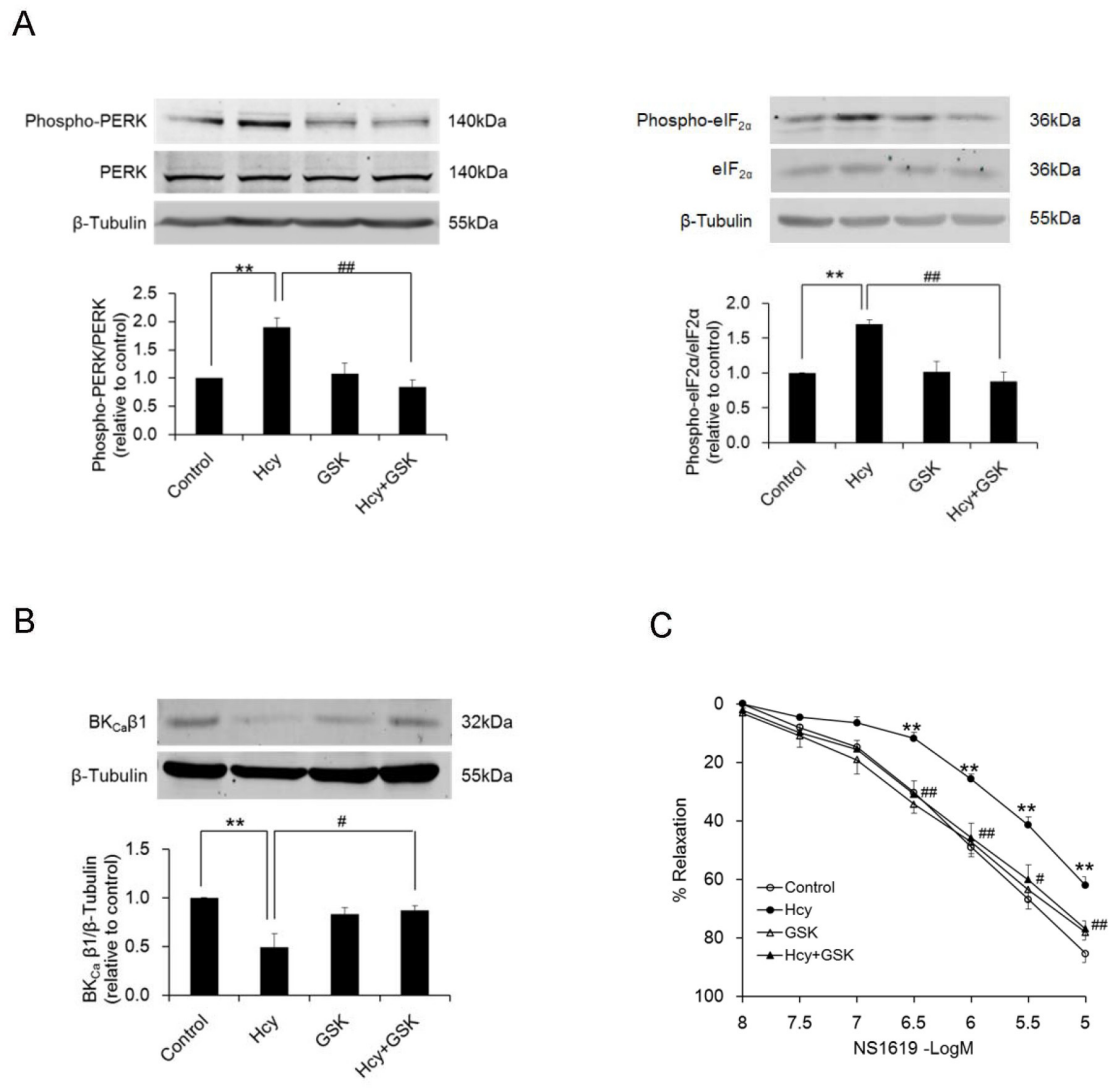
Inhibition of activation of PERK branch of ER stress by GSK2606414, evidenced by decreased phosphorylation of PERK and eIF2α (A, n=4), prevents homocysteine-induced loss of BK_Ca_ β1 protein in PCASMCs (B, n=4), which is accompanied by an improved vasorelaxant response to NS1619 (C, n=8) **p<0.01 vs. control; ^#^p<0.05, ^##^p<0.01 vs. Hcy. N indicates the number of independent experiments, each obtained from cell isolates (a & b) or vessels (c) of different heart. Hcy: homocysteine, GSK: GSK2606414.

**Figure 4:**
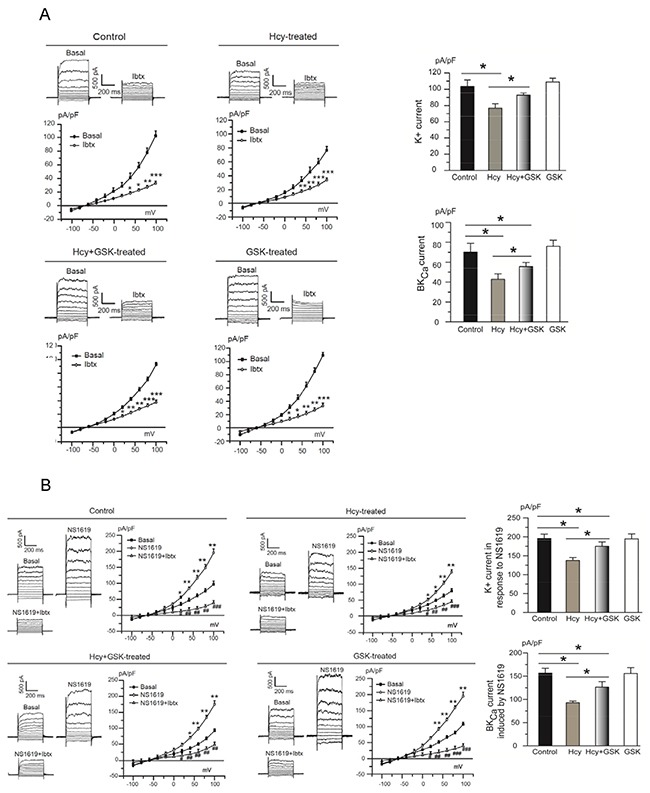
Homocysteine exposure suppresses BK_Ca_ channel currents in PCASMCs Treatment of the cells with the selective PERK inhibitor GSK2606414 protects BK_Ca_ channels against homocysteine-induced inhibition. Original traces and I-V curves of whole-cell K^+^ current under unstimulated condition (**A - *left panel***) and in response to NS1619 (**B - *left panel***) in cells from different treatment groups before and after application of the BK_Ca_ channel blocker iberiotoxin. *p<0.05, **p<0.01, ***p<0.001 vs. basal, ^#^p<0.05, ^##^p<0.01, ^###^p<0.001 NS1619+Ibtx vs. NS1619. Summarized data of BK_Ca_ channel current from 5 independent experiments under conditions without (**A - *right panel***) or with NS1619 stimulation (**B - *right panel***), each obtained from cell isolates of different heart. *p<0.05. Hcy: homocysteine, GSK: GSK2606414, Ibtx: iberiotoxin.

The selective PERK inhibitor GSK2606414 effectively suppressed the activation of PERK branch of ER stress in homocysteine-exposed PCASMCs. Phosphorylation of PERK and its downstream molecule eIF2α was inhibited (Figure 3A). Inhibition of PERK activation with GSK2606414 preserved the relaxant response to NS1619 in PCAs subjected to homocysteine exposure. The maximal response was 77.0±2.8% in homocysteine-exposed arteries with GSK2606414 treatment, in comparison with 62.2±3.0% in those without GSK2606414 treatment (Figure 3C). Further patch clamp recording showed that inhibition of PERK protects smooth muscle BK_Ca_ channels from homocysteine-induced inhibition. Both basal K^+^ current (76.74±5.30 vs. 103.37±7.99 pA/pF in control, p<0.05) and the current in response to NS1619 (137.57±7.67 vs. 195.59±11.59 pA/pF in control, p<0.05) (Figure 4A & 4B) were significantly suppressed in PCASMCs subjected to homocysteine exposure, which largely resulted from BK_Ca_ channel inhibition. Differentiation of BK_Ca_ component with the specific BK_Ca_ channel blocker iberiotoxin showed that after homocysteine exposure basal BK_Ca_ channel current decreases from 70.07±8.69 pA/pF to 42.83±5.50 pA/pF (p<0.05) and the BK_Ca_ current in response to NS1619 declines from 156.62±10.34 pA/pF to 92.76±3.65 pA/pF (p<0.05) (Figure 4A & 4B). Compared with homocysteine-exposed PCASMCs, cells exposed to homocysteine and co-treated with GSK2606414 exhibited significant (p<0.05) larger current density of BK_Ca_ channels under both basal (55.46±4.32 pA/pF) and NS1619-stimulated (126.37±11.89 pA/pF) conditions (Figure 4A & 4B). The enhancement of BK_Ca_ channel current by GSK2606414 in homocysteine-exposed PCASMCs was observed to be associated with restoration of the protein level of BK_Ca_ β1 (Figure 3B). These data indicated that the PERK branch of ER stress mediates homocysteine-induced BK_Ca_ channel inhibition, which is attributed to the loss of β1 subunit of the channel.

### Activation of PERK branch of ER stress promotes nuclear translocation of FoxO3a in homocysteine-exposed PCASMCs

**Figure 5:**
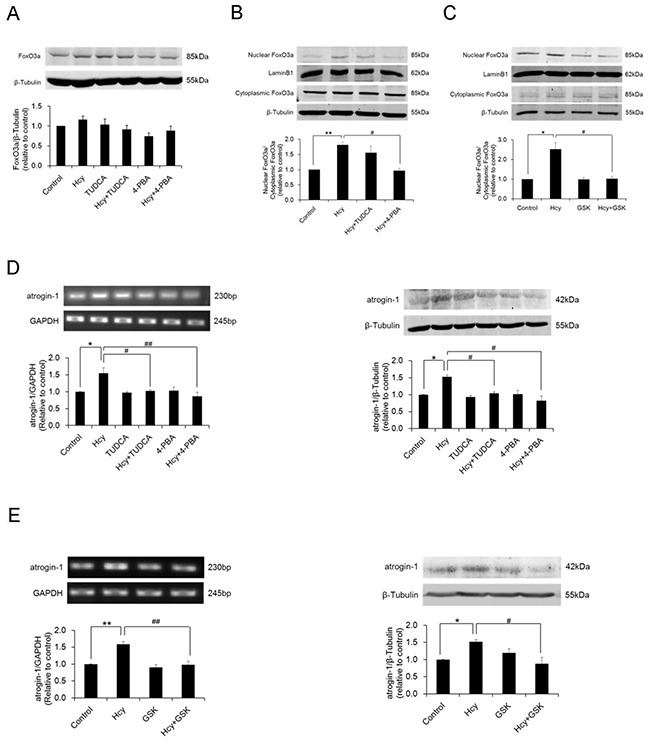
Homocysteine exposure promotes nuclear translocation of FoxO3a and upregulates atrogin-1 expression in PCASMCs While the overall level of FoxO3a expression remains unchanged (**A**, n=7), nuclear to cytoplasmic expression ratio of FoxO3a significantly increases in homocysteine-exposed PCASMCs (**B**, n=4). Inhibition of ER stress and selective blockade of the PERK branch of ER stress both effectively inhibit homocysteine-induced FoxO3a nuclear translocation (**B** & **C**, n=4) and atrogin-1 upregulation (**D** & **E**, n=4). *p<0.05, ^#^p<0.05; **p<0.01, ^##^p<0.01. Hcy: homocysteine, TUDCA and 4-PBA: ER stress inhibitors, GSK: GSK2606414, PERK selective blocker.

Homocysteine promoted the translocation of FoxO3a from cytoplasm to nucleus in PCASMCs. While the whole cell protein level of FoxO3a remained unchanged (Figure 5A), the nuclear/cytoplasmic expression ratio of FoxO3a significantly increased in PCASMCs subjected to homocysteine exposure (p<0.01 vs. control). Inhibition of ER stress prevented the increase of nuclear/cytoplasmic FoxO3a ratio induced by homocysteine (Figure 5B). Further experiments showed that activation of the PERK branch of ER stress drives nuclear translocation of FoxO3a in homocysteine exposure. As compared with homocysteine-exposed PCASMCs, cells co-treated with homocysteine and the PERK inhibitor GSK2606414 showed significantly reduced nuclear/cytoplasmic expression ratio of FoxO3a, which does not differ from that of the cells without homocysteine exposure (Figure 5C).

### Activation of FoxO3a by PERK-ER stress signaling mediates homocysteine-induced loss of BK_Ca_ β1 via upregulation of atrogin-1

Homocysteine exposure increased mRNA and protein expressions of atrogin-1 in PCASMCs, which were prevented by co-incubation of the cells with ER stress inhibitors TUDCA and 4-PBA (Figure 5D). Prevention of atrogin-1 upregulation was also achieved by the PERK selective inhibitor GSK2606414 (Figure 5E). These results suggest that activation of PERK pathway of ER stress is responsible for homocysteine-induced atrogin-1 expression in PCASMCs.

**Figure 6:**
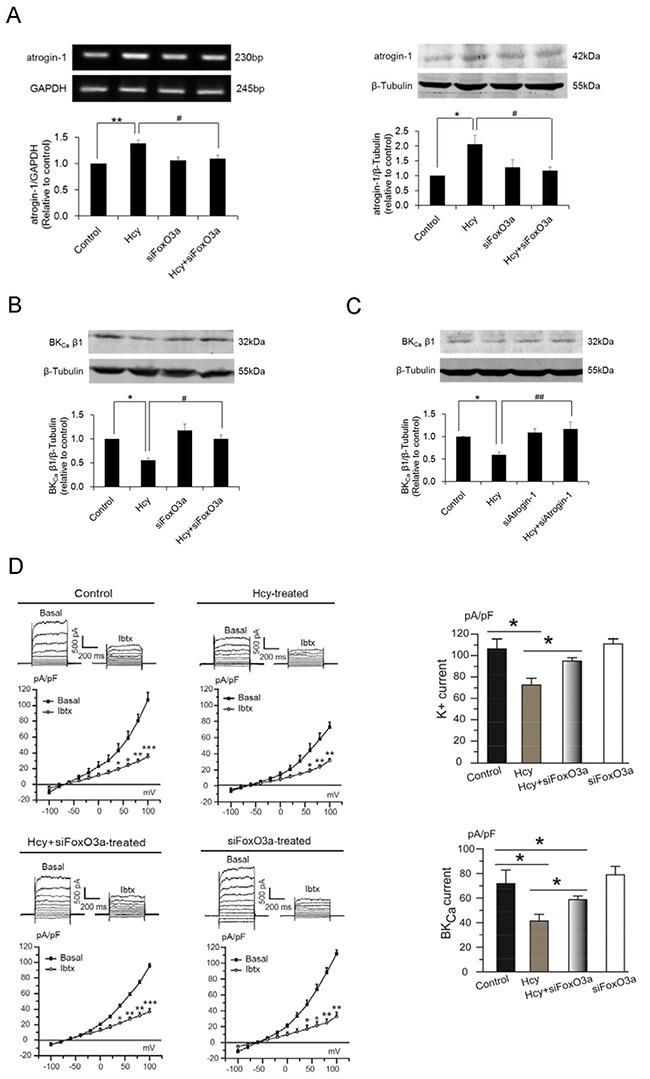

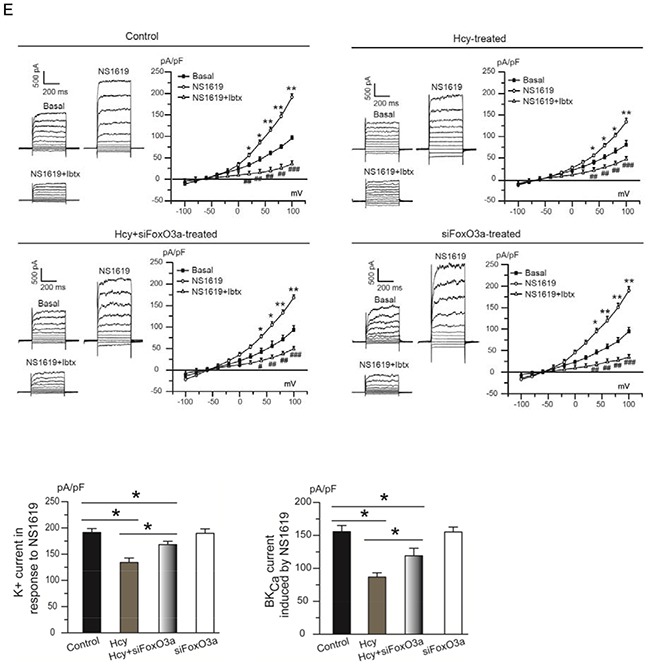
Silencing of the FoxO3a gene by siRNA prevents atrogin-1 upregulation **(A)** and BK_Ca_ β1 downregulation **(B)** in homocysteine-exposed PCASMCs. The role of atrogin-1 as a critical mediator of homocysteine-induced BK_Ca_ β1 loss is evidenced by the preserved protein level of BK_Ca_ β1 in homocysteine-exposed cells that are transfected with atrogin-1 siRNA **(C)**. *p<0.05, **p<0.01; ^#^p<0.05, ^##^p<0.01. Silencing of the FoxO3a gene prevents homocysteine-induced inhibition of BK_Ca_ channel currents (**D** & **E**). Original traces and I-V curves of whole-cell K^+^ current under unstimulated condition (D - left panel) and in response to NS1619 (E - left panel) in cells from different treatment groups before and after application of the BK_Ca_ channel blocker iberiotoxin. *p<0.05, **p<0.01, ***p<0.001 vs. basal, ^#^p<0.05, ^##^p<0.01, ^###^p<0.001 NS1619+Ibtx vs. NS1619. Summarized data of BK_Ca_ channel currents from 5 independent experiments under conditions without (D - right panel) or with NS1619 stimulation. (E - lower panel), each obtained from cell isolates of different heart. *p<0.05. Hcy: homocysteine, Ibtx: iberiotoxin.

Silencing of FoxO3a with siRNA normalized atrogin-1 expression in homocysteine-exposed PCASMCs (Figure 6A). Meanwhile, FoxO3a knockdown restored homocysteine-induced reduction of BK_Ca_ β1 protein (Figure 6B), with consequent enhancement of BK_Ca_ channel currents. Compared with homocysteine-exposed control cells, basal BK_Ca_ currents increased from 41.37±5.39 pA/pF to 58.77±2.92 pA/pF and BK_Ca_ currents activated by NS1619 enhanced from 86.77±6.49 pA/pF to 119.19±11.25 pA/pF in homocysteine-exposed cells that transfected with FoxO3a siRNA (Figure 6D & 6E). These results in conjunction with the finding of preserved BK_Ca_ β1 protein level in atrogin-1-silenced PCASMCs therefore unraveled atrogin-1 as a critical link between FoxO3a activation and BK_Ca_ β1 loss in homocysteine-induced BK_Ca_ channel dysfunction (Figure 6C).

## DISCUSSION

The present study for the first time demonstrated that in coronary arteries 1) ER stress mediates homocysteine-induced smooth muscle BK_Ca_ channel dysfunction; 2) loss of β1 subunit of BK_Ca_ triggered by the activation of PERK branch of ER stress is an underlying basis of the channel inhibition caused by homocysteine; 3) PERK-dependent activation of FoxO3a is responsible for the BK_Ca_ β1 loss and atrogin-1 acts as a critical downstream mediator of FoxO3a.

The ER of vascular cells becomes stressed when subjected to homocysteine exposure. As we previously observed in endothelial cells [22], homocysteine also induced ER stress in smooth muscle of coronary arteries. All three branches of the unfolded protein response (UPR), i.e., PERK, IRE1, and ATF6, were activated in homocysteine-exposed PCASMCs. Homocysteine caused significant loss of vasodilatory function of BK_Ca_ channels in endothelium-denuded coronary arteries and such loss was prevented by ER stress inhibitors, suggesting the role of ER stress in homocysteine-induced smooth muscle BK_Ca_ channel inhibition. Relaxation studies in arteries exposed to tunicamycin, a chemical ER stress inducer, provided parallel evidence that BK_Ca_ channels are susceptible to ER stress.

Further studies of channel expression demonstrated that decrease of β1 subunit resulting from ER stress is responsible for homocysteine-induced loss of vasodilatory function of BK_Ca_ channels. The β1 subunit has been well documented for its role in maintaining normal BK_Ca_ channel function in vasculature. Mice lacking BK_Ca_ β1 exhibits vascular smooth muscle hypercontractility and elevated blood pressure [28, 29]. In type 2 diabetes mellitus, downregulation of BK_Ca_ β1 was demonstrated to be a contributor to enhanced arterial tone [30]. The present study added one more piece of evidence supporting the significance of the β1 subunit of BK_Ca_ channels in vascular pathophysiology. More importantly, we for the first time demonstrated that ER stress is a key mediator of homocysteine-induced loss of BK_Ca_ β1, which provided new mechanistic insight into hyperhomocysteinemia-associated vascular dysfunction.

Protein homeostasis is maintained by a balanced regulation between synthesis and degradation. The reduced protein level whereas unchanged mRNA content of BK_Ca_ β1 following exposure to homocysteine indicates posttranscriptional regulation of the β1 subunit that might involve enhanced degradation of BK_Ca_ β1 protein. As a dominant mechanism in mammalian cells that controls the protein degradation, the ubiquitin-proteasome system is composed of an enzyme cascade involving E1 (ubiquitin-activating enzyme), E2 (ubiquitin-conjugating enzyme), and E3 (ubiquitin ligase) that facilitate the ubiquitination of the target protein for subsequent destruction by the proteasome [31]. Atrogin-1 (FbxO-32) is a muscle specific E3 ubiquitin ligase [32] that belongs to F-box only (FbxO) protein family [33]. The ability of atrogin-1 binding to the PDZ-binding motif in substrates [32] makes it potentially important in BK_Ca_ protein regulation since the PDZ-binding motif is present in most BK_Ca_ β_1_ isoforms in different species including human [26]. In fact, previous investigation in animal model of diabetes demonstrated that atrogin-1 facilitates BK_Ca_ β_1_ degradation in vascular smooth muscle cells [26]. In this study we observed that loss of BK_Ca_ β_1_ caused by homocysteine is also associated with an upregulation of atrogin-1, which suggests the role of E3 ubiquitin ligase in hyperhomocysteinemia-related vascular pathology.

FbxO expression is positively regulated by FoxOs [34]. In studies of diabetes, Zhang and colleges for the first time attributed atrogin-1-promoted BK_Ca_ β_1_ downregulation to FoxO activation in vascular smooth muscle cells [26]. We observed that in homocysteine-exposed PCASMCs, enhanced expression of atrogin-1 is associated with increased nuclear to cytoplasmic expression ratio of FoxO3a that implies increased FoxO3a transcriptional activity. siRNA knockdown of FoxO3a prevented homocysteine-induced atrogin-1 upregulation. In conjunction with the findings that silencing of FoxO3a or atrogin-1 normalized the protein level of BK_Ca_ β_1_ in homocysteine-exposed PCASMCs, these data provided additional proof of FoxO/atrogin-1 cascade in BK_Ca_ β_1_ regulation. The findings of restoration of BK_Ca_ channel currents by FoxO3a silencing further confirmed the significance of FoxO3a in homocysteine-induced BK_Ca_ channel inhibition.

Homocysteine-induced nuclear accumulation of FoxO3a and upregulation of atrogin-1 in PCASMCs were prevented by ER stress inhibitors and selective PERK inhibitor, suggesting that activation of the PERK branch of the UPR in response to ER stress stands upstream of FoxO3a activation. Our finding of PERK-dependent FoxO3a activation is in agreement with the report in human lung carcinoma cell line that depletion of PERK limits FoxO activity [35]. The essential role of PERK in FoxO/atrogin-1 activation therefore explains the capacity of the PERK inhibitor in restoring homocysteine-induced loss of β_1_ subunit of BK_Ca_ and the resulting enhancement of channel currents.

### Limitations of the study

By uncovering the role of ER stress in BK_Ca_ channel inhibition, we identified a new mechanistic link between hyperhomocystinemia and vascular dysfunction that is characterized as PERK-FoxO3a-atrogin-1-BK_Ca_ β1 regulation. Although our results demonstrated that activation of PERK branch of the UPR increases nuclear localization of FoxO3a thereby enhancing its activity to promote atrogin-1-mediated BK_Ca_ β1 degradation, how PERK activation facilitates nuclear translocation of FoxO3a was not further elucidated. Akt-mediated phosphorylation of FoxO is known as an important regulatory mechanism controlling the subcellular localization of FoxO [36], whether Akt is a possible mediator of PERK-induced FoxO3a activation in our study subject warrants further investigation. Another issue worthy of attention is that although inhibition of PERK and silencing of FoxO3a both restored the protein level of BK_Ca_ β1 in homocysteine-exposed PCASMCs, these treatments were unable to fully recover the whole-cell BK_Ca_ channel currents. These results implied that mechanisms other than PERK-FoxO3a-mediated loss of β1 subunit may also take part in homocysteine-induced inhibition of BK_Ca_ channels. One potential possibility is inhibition of the activity of single BK_Ca_ channel. It will be interesting to further study whether PERK and/or the other two UPR branches, i.e. IRE1 and ATF6 have an impact on this by employing single-channel recording techniques. Moreover, in light of the evidence that ROS virtually eliminates activation of single BK_Ca_ channel by targeting a cysteine residue near the Ca^2+^ bowl of the BK_Ca_ α subunit [37], and the cross-talk between ER stress and oxidative stress [38], future studies aiming at signaling cascades linking ER stress and ROS may help provide profound knowledge on the role of ER stress in vascular BK_Ca_ channel dysfunction in hyperhomocysteinemia.

In conclusion, the present study demonstrated that homocysteine impairs coronary dilator function through a mechanism involving ER stress-mediated inhibition of smooth muscle BK_Ca_ channels. Atrogin-1-promoted degradation of BK_Ca_ β1 subunit plays a key role in the ER stress-mediated channel inhibition that could be attributed to PERK-dependent FoxO3a activation. This study provides a new understanding of the molecular mechanisms involved in homocysteine-induced vascular injury and offers new perspectives in the development of vascular protection strategies for patients with hyperhomocystinemia.

## MATERIALS AND METHODS

Fresh hearts of young adult pigs (~35 kg) were collected from a local slaughterhouse. Once excised, the hearts were placed in cold (4°C), pre-oxygenated (95%O_2_/5%CO_2_) Krebs solution and immediately transferred to the laboratory for dissection of small coronary arteries as in our previous studies [15, 39, 40]. All experiments were in accordance with institutional guidelines. The study was approved by the Animal Experimentation Ethics Committee, and Safety Office of the Chinese University of Hong Kong (Ref. No. 14/010/GRF).

### Isolation and primary culture of porcine coronary artery smooth muscle cells (PCASMCs)

PCASMCs were cultured after isolation by enzymatic digestion from porcine small coronary arteries [41]. In brief, small coronary arteries were cut open longitudinally and the endothelium was gently scraped off with a microsurgical blade. The tissue strip was then washed with cold Krebs and dissected into 1×1 mm^2^ strips, followed by 30-min digestion at 37°C in 2 ml of phosphate-buffered saline solution (PBS), containing 2 mg/ml of collagenase (type 2; Worthington Biochemicals, USA), 0.5 mg/ml of papain (Worthington Biochemicals, USA), 1.75 mg/ml of DL-Dithiothreitol (DTT, Sigma, USA), and 5 mg/ml albumin bovine (BSA, Amresco, USA). The enzymatic activity was stopped by Dulbecco's Modified Eagle Medium (DMEM, Thermo Fisher Scientific, USA) containing 10% fetal bovine serum (FBS, Thermo Fisher Scientific, USA). The suspension was centrifuged at 1600 rpm for 5 min. Cells were then resuspended in 12 ml DMEM containing 10% FBS, 100 U/ml penicillin, and 100 μg/ml streptomycin (Thermo Fisher Scientific, USA). After 1-h incubation at 37°C, the medium was replaced once to remove unattached cells. Attached PCASMCs were cultured in a humidified incubator with 5%CO_2_ at 37°C. For maintaining electrophysiological properties of isolated PCASMCs, only primary cultured cells were used for experiments.

### Patch-clamp recording of BK_Ca_ channel currents in PCASMCs

Whole-cell K^+^ current of PCASMCs were recorded by patch-clamp (EPC10, HEKA, Lambrecht, Germany) at room temperature (20-24°C) with further differentiation of the BK_Ca_ component [41]. Patch pipettes with resistance of 3-5 MΩ were filled with a solution containing (mmol/L): NaCl 10, KCl 110, MgCl_2_ 4, CaCl_2_ 7, ethylene-glycol tetraacetic acid (EGTA) 10, Mg-ATP 5, and HEPES 10. The bath solution contained (mmol/L): NaCl 130, KCl 5, MgCl_2_ 1.2, CaCl_2_ 1.5, Glucose 10, and HEPES 10. PCASMCs were held at -60 mV and voltage steps ranging from -100 to +100 mV were applied for 500 milliseconds in 20-mV step increments. K^+^ currents without and with stimulation of the BK_Ca_ channel activator NS1619 (3 μmol/L) were recorded with further application of the specific BK_Ca_ blocker iberiotoxin (100 nmol/L) to identify the BK_Ca_ current. Data were analyzed with PulseFit software (HEKA). The current was normalized by cell capacitance into current densities (pA/pF).

### Isometric force study

Porcine small coronary arteries (PCAs) (300-500 μm in diameter) were dissected from the branches of left anterior descending artery (LAD) and cut into cylindrical rings with 2-mm in length. Prior to mounting, normalization, and equilibration in a four-channel Mulvany myograph (Model 610M, J.P.Trading, Aarhus, Denmark) as published elsewhere [15, 40], the rings were denuded of endothelium by gently rubbing the intraluminal surface with wires and successful removal of endothelium was confirmed by the lack of relaxant response to the endothelium-dependent vasodilators, as in our previous studies [42, 43]. Cumulative dose-response curve to the BK_Ca_ activator NS1619 (-8~-5 Log M) were then established following U46619 pre-contraction in the endothelium-denuded rings.

### Reverse-transcriptase polymerase chain reaction (RT-PCR) analysis

Total RNA from PCASMCs was extracted using total RNA reagent (RNAiso Plus, TaKaRa Biotechnology, China) and mRNA was converted to cDNA using reverse transcriptase kit (PrimeScript™ RT Master Mix, TaKaRa Biotechnology, China) according to manufacturer's instructions. PCR amplification was performed with Taq DNA polymerase (GoTaq® G2 Flexi DNA Polymerase, Promega, USA) and the thermal cycling conditions were: 95°C for 30s, followed by 30 cycles of 95°C for 30s, 50°C for 1 min, 72°C for 1 min, and completed by 72°C for 5 min. PCR products were then separated by 1.5% agarose gel electrophoresis. Density analysis of the signals was evaluated using the GelQuant.NET 1.8.2 software (Biochem Lab Solutions, University of California, San Francisco). Primers used for PCR amplification were as follows: BK_Ca_ α, 5’-GCCAGCAACTTTCACTAC-3’ (forward) and 5’-CTGACAGGATAACGCACA-3’ (reverse); BK_Ca_ β1, 5’-TGTGCTGTCATCGCCTACT-3’ (forward) and 5’-ACCTGGTGCTCGTGGAAC-3’ (reverse); FoxO3a, 5’-ACATGGCCGGAACCATGAAT-3’ (forward) and 5’-GTCCAAACACTGTGCTGCTG-3’ (reverse); atrogin-1, 5’-TGGATGGCTGGGGATACAGA-3’ (forward) and 5’-TAAATTCCCCGCCAGTGTCC-3’ (reverse). GAPDH was amplified in parallel as an internal loading control with primers 5’-GGTCGGAGTGAACGGATTT-3’ (forward) and 5’-ATTTGATGTTGGCGGGAT-3’ (reverse).

### Western blot analysis

#### Protein expression at whole cell level

Whole cell lysates of PCASMCs were used for protein expression analysis of 1) α and β1 subunits of BK_Ca_ channels; 2) ER stress molecules including 78-kDa glucose regulated protein (GRP78), protein kinase RNA-like ER kinase (PERK), phosphorylated PERK, inositol-requiring enzyme 1 (IRE1), phosphorylated IRE1, activating transcription factor 6 (ATF6), eukaryotic translation initiation factor 2α (eIF2α) and phosphorylated eIF2α; 3) FoxO3a; and 4) atrogin-1.

Whole cell protein was extracted with lysis buffer containing protease and phosphatase inhibitor cocktail (Roche Diagnostic). The protein extraction was centrifuged at 4°C for 20 min at 12000 rpm and mixed with loading buffer, heated up to 100°C for 10 min, then fractionated by a denaturing 8% sodium-dodecyl-sulfate polyacrylamide-gel electrophoresis (30 μg per lane) for 90 min at 120V and electro-transferred to polyvinylidene difluoride (PVDF) membrane (Thermo Scientific) for 90 min at 100V. The membrane was blocked with 5% non-fat milk/TBST for 1 h at room temperature and incubated with the primary antibody in 5% non-fat milk/TBST overnight at 4°C. Primary antibodies used include BK_Ca_ α (1:1000 dilution, Abcam), BK_Ca_ β1 (1:500, Abcam), GRP78 (1:1000, Abcam), PERK (1:1000, Cell Signaling), phosphorylated (Thr980) PERK (1:500, Bioss), IRE1 (1:1000, Abcam), phosphorylated (Ser724) IRE1 (1:1000, Abcam), eIF2α (1:1000, Abcam), phosphorylated (Ser51) eIF2α (1:500, Abcam), ATF6 (1:1000, Abnova), FoxO3a (1:500, Abcam), and atrogin-1 (1:500, Abcam). The membrane was then washed in TBST followed by incubation with secondary IRDye800®-infrared fluorescent dye-conjugated goat anti-rabbit or rabbit anti-mouse antibody (1:10000, Rockland) in TBST for 1 h at room temperature. Imaging was performed at a wavelength of 800 nm by using Odyssey gel imaging scanner (Li-Cor Biosciences). The intensity of the bands was analyzed by Quantity One imaging system (Version 4.6.6, Bio-Rad) and β-tubulin (1:2500, Abcam) was used as internal loading control.

#### Protein expression at subcellular level

The NE-PER™ Nuclear and Cytoplasmic Extraction Reagents (Thermo Fisher Scientific, USA) were used to separate nuclear and cytoplasmic fractions of PCASMCs. Briefly, primary cultured PCASMCs were harvested with trypsin-EDTA (Thermo Fisher Scientific, USA) and centrifuged at 500×g for 5 min to collect the cell pellet. After wash in PBS, cells were centrifuged to remove the wash buffer and ice-cold cytoplasmic extraction reagents I and II were then added to the cell pellet in proportion of 200:11 (μl). The supernatant was collected as cytoplasmic lysate after 5-min centrifugation at 16,000×g. Nuclear extraction reagent (100 μl) was then added to the precipitate for further centrifugation (16,000×g, 10 min) to collect the supernatant as the nuclear lysate. Western blotting was then performed using proteins from nuclear and cytoplasmic preparations for expression analysis of FoxO3a. Lamin B1 (1:2000, Abcam) and β-tubulin (1:2500, Abcam) were used as nuclear and cytoplasmic loading control respectively.

### Gene silencing by siRNAs

Primary cultured PCASMCs were transfected with specific siRNA targeting porcine FoxO3a and atrogin-1, respectively. For each target, three pairs of siRNAs were designed along with a scramb-led control siRNA: siFoxO3a-1: 5’-CCGGCUGGA AGAACUCUAUTT-3’ (sense), 5’-AUAGAGUUCUU CCAGCCGGTT-3’(antisense); siFoxO3a-2: 5’-CCGGA ACCAUGAAUCUCAATT-3’ (sense), 5’-UUGAGAUUC AUGGUUCCGGTT-3’ (antisense); siFoxO3a-3: 5’-GG AGCUUGGAAUGUGACAUTT-3’ (sense), 5’-AUGUC ACAUUCCAAGCUCCTT-3’ (antisense); siAtrogin-1-1, 5’-CCUUCAAAGGCCUCACCUUTT-3’ (sense), 5’-AAG GUGAGGCCUUUGAAGGTT-3’ (antisense); siAtrogin-1 -2, 5’-GCAACUGAACAUCAUGCAATT-3’ (sense), 5’-UUGCAUGAUGUUCAGUUGCTT-3’ (antisense); siAtrogin-1-3, 5’-GCCACAUUCUUUCCUGGAATT-3’ (sense), 5’-UUCCAGGAAAGAAUGUGGCTT-3’ (antisense). To perform transfection, PCASMCs were seeded in 6-well plate and cultured in DMEM containing 10% FBS, 100 U/ml penicillin, and 100 μg/ml streptomycin until they reached 70-80% confluence. Cells were then incubated in Opti-MEM® I Reduced Serum Media (Thermo Fisher Scientific, USA) for 24 h. The mixture of siRNA (gene-specific or corresponding scrambled control) (20 μmol/L, Shanghai GenePharma, China) and Lipofectamine® 2000 Transfection Reagent (Thermo Fisher Scientific, USA) in proportion of 5:2 was added with further incubation for 6 h. Culture medium was then changed to normal DMEM and siRNA knockdown efficiency was determined at both mRNA level by RT-PCR and protein level by western blotting after 24 and 48 h, respectively. For each target, the siRNA displaying the highest knockdown efficiency was chosen for subsequent experiments.

### Experimental protocols

### Studies of the role of ER stress in homocysteine-induced BK_Ca_ channel dysfunction

#### Effect of homocysteine on BK_Ca_ channel-mediated vasorelaxation – role of ER stress

PCA rings taken from the same branch of the LAD were scraped off endothelium before allocated to different treatment groups and treated for 24 h as: control, homocysteine (100 μmol/L), tauroursodeoxycholate (TUDCA, ER stress inhibitor, 200 mmol/L), homocysteine+TUDCA, 4-phenylbutyric acid (4-PBA, ER stress inhibitor, 2 mmol/L), homocysteine+4-PBA. After pre-constriction with U46619, vasorelaxant responses to the BK_Ca_ activator NS1619 (-8~-5 LogM) were studied.

#### Effect of ER stress inducer on BK_Ca_ channel-mediated vasorelaxation

Endothelium-denuded PCAs were allocated and treated for 4 h as: control, tunicamycin (chemical ER stress inducer, 5 μg/ml), TUDCA, tunicamycin+TUDCA, 4-PBA, tunicamycin+4-PBA. NS1619-induced vasorelaxation was then studied in U46619 pre-constriction.

### Studies of the effect of homocysteine on BK_Ca_ channel expression *–* role of ER stress

PCASMCs were divided into six groups and treated for 24 h as: control, homocysteine, TUDCA, homocysteine+TUDCA, 4-PBA, and homocysteine+4-PBA. Cells were then collected for determination of 1) protein expression and phosphorylation of ER stress molecules, i.e., GRP78, ATF6, p-PERK/PERK, p-eIF2α/eIF2α, and p-IRE1/IRE1; and 2) mRNA and protein expressions of α and β1 subunits of BK_Ca_.

### Studies of the role of PERK branch of ER stress in homocysteine-induced BK_Ca_ channel dysfunction

#### Effect of pharmacological inhibition of PERK on BK_Ca_ channel-mediated relaxation in homocysteine-exposed PCAs

Endothelium-denuded PCAs were allocated and treated for 24 h as: control, homocysteine, GSK2606414 (PERK selective inhibitor [44], Selleckchem, USA, 500 nmol/L), and homocysteine+GSK2606414. NS1619-induced relaxation was studied after U46619 preconstriction.

#### Effect of pharmacological inhibition of PERK on BK_Ca_ channel expression and BK_Ca_ current in homocysteine-exposed PCASMCs

PCASMCs were grouped and treated as above, followed by determination of protein levels of BK_Ca_, p-PERK/PERK, and p-eIF2α/eIF2α and recording of BK_Ca_ current.

### Studies of the effect of homocysteine on FoxO3a and atrogin-1 *–* role of PERK branch of ER stress

#### Role of ER stress in the regulation of FoxO3a and atrogin-1 in homocysteine-exposed PCASMCs

PCASMCs were allocated to the following treatment groups: control with or without 4-PBA or TUDCA, and homocysteine with or without 4-PBA or TUDCA. After 24 h, mRNA and protein expressions of atrogin-1, protein expression of FoxO3a, and FoxO3a protein level in both the nucleus and the cytoplasm were examined.

#### Role of PERK branch of ER stress in homocysteine-induced FoxO3a and atrogin-1 regulation

PCASMCs were treated for 24 has: control, homo-cysteine, GSK2606414, and homocysteine+GSK2606414, followed by determination of mRNA and protein expressions of atrogin-1 and nuclear/cytoplasmic expression of FoxO3a.

### Studies of the role and relations of FoxO3a and atrogin-1 in homocysteine-induced BK_Ca_ channel dysfunction

#### Effect of FoxO3a knockdown on atrogin-1 expression in homocysteine-exposed PCASMCs

PCASMCs were transfected with scrambled control or specific FoxO3a siRNA before subjected to 24-h exposure to homocysteine. mRNA and protein expressions of atrogin-1 were then examined.

#### Effect FoxO3a knockdown on BK_Ca_ channel expression and activity in homocysteine-exposed PCASMCs

PCASMCs transfected with scrambled control or specific FoxO3a siRNA were treated as above, followed by detection of protein expression and current recording of BK_Ca_ channels.

#### Effect of atrogin-1 knockdown on BK_Ca_ channel expression in homocysteine-exposed PCASMCs

PCASMCs were transfected with scrambled control or specific siRNA targeting atrogin-1 before subjected to 24-h exposure to homocysteine. Cells were then examined for BK_Ca_ protein level.

### Data analysis

Relaxation was expressed as the percentage decrease of pre-contraction induced by U46619. All data were presented as mean±s.e.m. Statistical analyses were performed by one-way ANOVA followed by Scheffe tests (SPSS, version 20). Differences were considered as significant at p<0.05.

## Abbreviations

4-PBA: 4-phenylbutyric acid
ATF6: activating transcription factor 6
BK_Ca_ channel: large-conductance Ca^2+^-activated K^+^ channel
eIF2α: eukaryotic translation initiation factor 2α
ER: endoplasmic reticulum
FoxO: forkhead box O transcription factor
GRP78: 78-kDa glucose regulated protein
IRE1: inositol-requiring enzyme 1
PCAs: porcine small coronary arteries
PCASMCs: porcine small coronary artery smooth muscle cells
PERK: protein kinase RNA-like ER kinase
ROS: reactive oxygen species
TUDCA: tauroursodeoxycholate
UPR: unfolded protein response
